# A Machine Learning-Based Framework for Risk Recognition and Reliability Evaluation in City Expressway Ramp Merging

**DOI:** 10.3390/s26092779

**Published:** 2026-04-29

**Authors:** Zimu Li, Sheng Hu, Ke Zhang

**Affiliations:** 1 College of Traffic Transportation, Chongqing Jiaotong University, Chongqing 400074, China; 2 School of Artificial Intelligence and Big Data, Chongqing Industry Polytechnic University, Chongqing 401120, China; 3 China Waterborne Transport Research Institute, Beijing 100088, China

**Keywords:** traffic risk recognition, machine learning, UAV trajectory data, ramp merging, surrogate safety measures

## Abstract

Risk identification in ramp merging is often compromised by complex vehicle interactions and ‘future information’ leakage. To resolve this, we decouple the process using an ‘observation-conflict’ mechanism. By extracting kinematic features solely from the merging preparation phase, the framework predicts continuous risks during actual execution without temporal bias. Furthermore, we stabilize risk detection by integrating cpTTC thresholds with duration constraints into a three-level risk labeling scheme, ensuring the results align with real-world physical dynamics. Multiple machine learning models are comparatively evaluated using group-based data partitioning. Results indicate that the XGBoost model achieves the best overall performance, yielding an overall accuracy of 0.8182 and a multiclass AUC (OvR) of 0.8898. Furthermore, time cross-domain validation under varying macroscopic traffic flow states demonstrates that the framework exhibits reliable statistical stability; when reconstructed into a binary classification task, it maintains a risk recall of 0.9978. These findings provide a reliable methodological basis and early-warning feature reference for dynamic traffic risk assessment in merging scenarios.

## 1. Introduction

### 1.1. Background and Motivation

Ramp merging is one of the most common and high-risk traffic scenarios in city expressways and mainline traffic systems. In this scenario, vehicles must perform a series of continuous decisions, including speed adjustment, gap judgment, and path selection within a limited distance, while interactions among multiple vehicles are highly dynamic and inherently uncertain. When traffic demand increases or vehicle density rises, the safety gaps between vehicles can quickly narrow, causing the risk state to change significantly in a short time. Therefore, timely identification of potential risks under complex interactive conditions has long been an important issue in traffic safety analysis and the design of active safety systems.

With the development of drone-based aerial observation and high-precision trajectory extraction techniques, researchers can continuously record vehicle movements at the microscopic level and quantify traffic risks using surrogate safety indicators or data-driven models. Compared with traditional accident-based analysis, these approaches enable continuous monitoring of vehicle interactions and provide richer behavioral information for risk assessment. In recent years, machine learning and statistical modeling techniques have further expanded the analytical capability of traffic safety research, allowing complex nonlinear relationships among traffic variables to be captured more effectively.

Meanwhile, several emerging research directions have focused on improving the representation and interpretation of dynamic risk processes. Event-oriented analytical approaches, such as event-history analysis [1], have been introduced to describe temporal evolution patterns in complex systems, emphasizing that risk development is a cumulative process rather than a single-time-point outcome. In addition, dimensionality reduction and feature aggregation methods have been widely applied to simplify high-dimensional trajectory data and improve computational efficiency. Furthermore, multi-criteria decision-making frameworks have been adopted to evaluate and rank risk states based on multiple indicators, enabling more structured decision support in traffic safety management [2].

Despite these advances, several fundamental challenges remain in the practical deployment of data-driven risk identification methods. First, many existing models rely on input variables that may implicitly include post-decision information or future system responses. This inconsistency between model input and real decision conditions can lead to information leakage during training, resulting in overly optimistic performance estimates and reduced reliability in real-world applications. Second, risk labels are often defined using instantaneous threshold conditions, which may cause instability in classification results when traffic states fluctuate over short time periods. Third, model validation is frequently conducted under a single dataset or random sampling strategy, making it difficult to assess the robustness and generalization ability of risk identification results across different traffic environments.

To address these limitations, this study proposes a risk identification method framework designed to align model construction with real decision-making processes. The framework introduces consistency constraints in three key stages: feature construction, risk definition and model verification. During feature construction, only trajectory information observable before the decision moment is used to ensure that model inputs reflect realistic operational conditions. During risk definition, duration-based constraints are incorporated to improve the temporal stability of risk states. During model validation, a group-based data partitioning strategy and cross-environment verification mechanism are adopted to evaluate the robustness and transferability of model performance under varying data conditions.

Through these mechanisms, the proposed framework emphasizes decision-consistent modeling rather than solely improving prediction accuracy, aiming to enhance the credibility, stability, and deployability of risk identification results in complex traffic interaction scenarios, with potential applications in vehicle–infrastructure cooperative systems, where the method can function as a perception and risk assessment module to support proactive safety interventions such as early warning, cooperative speed adjustment, and coordinated braking.

### 1.2. Literature Review

In recent years, with the rapid development of traffic data collection technologies and data-driven methods, traffic risk identification based on vehicle microscopic trajectory data has gradually become an important direction in traffic safety research. Existing studies mainly focus on the construction of surrogate safety indicators, data-driven risk identification methods, applications of high-precision trajectory data, and model generalization capability, and are gradually moving toward more refined and dynamic risk identification.

#### 1.2.1. Risk Characterization Based on Surrogate Safety Measures

Surrogate Safety Measures (SSM) are widely used tools for risk characterization in traffic safety research. Their core idea is to identify potential conflict risks by analyzing the relative motion states of vehicles when real accident data are scarce. Hayward (1972) first proposed a method for quantifying near-miss events based on risk levels [3]. Later, Minderhoud and Bovy (2001) extended the Time-to-Collision (TTC) metric, providing a foundation for the subsequent development of surrogate safety indicators [4].

With further research, SSM has been widely applied in traffic safety analysis and assessment. Mahmud et al. (2017) systematically reviewed the applications of various surrogate safety indicators, highlighting their ability to effectively reflect potential risk states in the absence of accident data [5]. Wang and Stamatiadis (2014) validated the feasibility of surrogate safety indicators for traffic safety assessment using simulation data [6]. Additionally, Astarita et al. (2019), Montella (2010), and Nikolaou et al. (2023) expanded the application of surrogate safety indicators from perspectives such as model comparison, accident hotspot identification, and accident data analysis [7,8,9]. Overall, surrogate safety indicators have become essential tools in traffic safety research, but single-time metrics often fail to fully capture the dynamic changes during vehicle interactions. Therefore, incorporating continuous time-series information for more stable risk characterization has gradually become a key research direction in this field.

#### 1.2.2. Traffic Risk Identification Based on Machine Learning

With the continuous growth of traffic data, machine learning methods have been increasingly applied in traffic safety research to identify potential risk patterns in complex traffic environments. Arbabzadeh and Jafari (2017) proposed a risk prediction method based on driving behavior and road information data, demonstrating the feasibility of data-driven approaches in traffic safety modeling [10]. Lu et al. (2020) employed gradient boosting models for traffic accident prediction, showing that ensemble learning methods can effectively handle the nonlinear relationships in traffic data [11].

In recent years, deep learning methods have gradually become an important technical path for traffic risk identification research. Rahim and Hassan (2021) proposed a traffic accident severity prediction framework based on deep learning [12]; Shangguan et al. (2021) built a real-time driving risk prediction model based on natural driving data [13]; Hu et al. (2022) advanced this line of research by employing high-precision trajectory data to perform real-time traffic safety assessment [14]. In addition, Cai et al. (2022) combined street view data analysis of the impact of the driving environment on traffic safety [15], Hussain et al. (2025) proposed a real-time accident risk prediction method based on machine learning and verified its application potential in complex traffic scenarios [16]. In general, data-driven methods can effectively explore the potential risk patterns in traffic data, and gradually become an important technical path for traffic safety research [17,18].

#### 1.2.3. Application of High-Precision Trajectory Data in Traffic Behavior and Risk Research

The development of high-precision trajectory data provides an important data basis for the study of vehicle behavior and traffic risks. In recent years, a variety of public trajectory datasets have been widely used in traffic behavior analysis and risk identification research, for example, the highD dataset [19] proposed by Krajewski et al. (2018), the inD dataset [20] released by Moers et al. (2019), and the rounD dataset [21] proposed by Bock et al. (2020). They all provide high-quality data support for the study of vehicle interaction behavior in complex traffic scenarios.

At the same time, the early trajectory dataset also plays an important role in the study of traffic behavior. Coifman and Li (2017) conducted a systematic evaluation of the NGSIM dataset [22], Raju et al. (2018) used trajectory data to analyze the behavior characteristics of vehicles in a mixed traffic environment [23], and Yuan et al. (2022) further based on traffic flow Features propose a real-time conflict risk prediction method [24]. In general, with the continuous improvement of trajectory data accuracy and data scale, traffic safety research has gradually shifted from static statistical analysis to dynamic behavior modeling based on continuous time series [25].

#### 1.2.4. Model Generalization Ability and Cross-Scene Applicability Issues

With the wide application of data-driven methods in the field of traffic safety, the stability and applicability of models in different data environments have gradually attracted attention. Pan and Yang (2009) systematically summarize the basic theory of migration learning and provide an important methodological basis for the application of cross-scene models [26]. In recent years, relevant studies have gradually applied migration learning and domain adaptive methods to the fields of traffic forecasting and safety analysis.

For example, Sengupta et al. (2024) proposed a Bayesian-based method to quantify uncertainty in traffic prediction models, highlighting that model generalization is a key factor affecting prediction performance [27]. Shao et al. (2025) proposed cross-city traffic prediction methods and demonstrated that domain adaptation techniques can effectively improve model applicability [28]. Hadjidimitriou et al. (2020) analyzed the applicability of data-driven models in different traffic systems from the perspective of demand–supply matching, noting that model performance may vary under different data conditions [29]. Therefore, in traffic risk identification research, relying solely on a single dataset or random sample partitions may not fully reflect the actual performance of a model. Existing evaluation methods also rarely consider the dynamic impact of traffic flow evolution across different time periods within the same scenario on the distribution of microscopic risk categories.

### 1.3. Research Gaps and Contributions of This Study

Existing studies have made certain progress in risk identification methods and data applications; however, in complex traffic interaction scenarios, the reliability and stability of models still face several common challenges. First, during feature construction, some studies do not strictly distinguish between pre-decision and post-decision information, which may allow models to access additional information during training, potentially overestimating their identification capability. Secondly, the definition of risk labels is often based on single threshold standards, which makes them more sensitive to short-term fluctuations in the state of the vehicle, resulting in insufficient label stability. Third, the model evaluation in many studies still relies mainly on a single dataset or random division method, and insufficient consideration is given to the performance of the model under different traffic conditions, which makes it difficult to fully reflect the stability and applicability of the model.

In order to solve the above problems, this study will focus on the ramp merger scenario and explore the key aspects of traffic risk identification, including reasonable data application, stability of risk state description and reliability of model prediction. The main contributions of this study are as follows:

- A decision-consistent interaction feature construction method is proposed, which clearly limits the input variable to only come from the observable state before decision-making, thus systematically eliminating the problem of information leakage in the process of risk identification and improving the credibility and deployability of the model.
- A multi-level risk definition method combined with term constraints has been developed. This method introduces time conditions into the risk determination process, thus improving the stability of risk labels in the time series and improving the consistency of model training and result interpretation.
- A set of data grouping and verification strategies has been formulated for different traffic conditions, so as to evaluate the applicability and robustness of the model under different traffic demand and density cycles.
- A unified risk identification framework has been built, which integrates feature consistency, label stability and model verification reliability, and demonstrates its effectiveness and stability through the application of actual UAV trajectory data in complex vehicle interaction scenarios.

## 2. Materials and Methods

**Figure 1:**
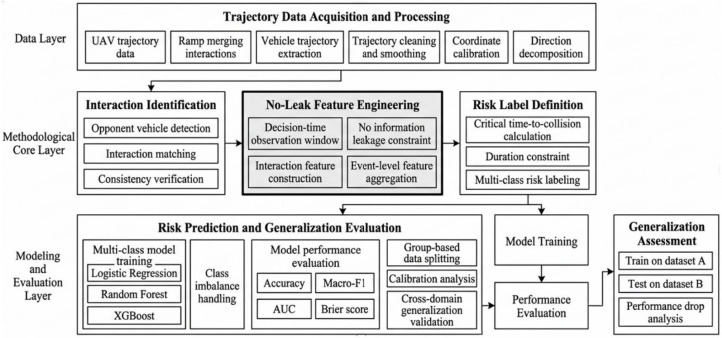
No-Leakage Risk Identification Framework.

Figure 1 presents the overall framework of the proposed methodology, covering data acquisition and processing, interaction feature engineering, risk label definition, and multi-model training with generalization evaluation. The framework specifies the decision-time observation window and no-information-leakage constraints, providing a foundation for subsequent experimental design and results analysis.

### 2.1. Trajectory Data Acquisition and Processing

#### 2.1.1. Trajectory Data Acquisition

**Figure 2:**
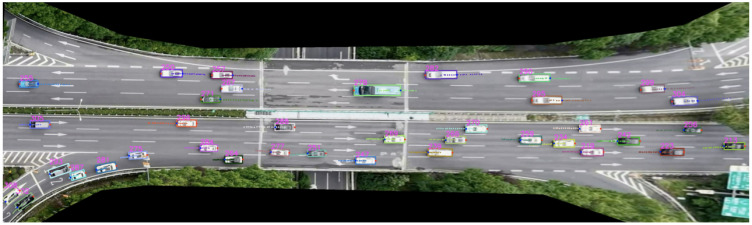
Extraction of Vehicle Trajectories from UAV Data.

Vehicle weaving trajectory data were collected using Unmanned Aerial Vehicle (UAV)-based aerial photography. Data acquisition was conducted during the evening peak hours on clear weekdays at the north–south weaving section of the Donghuan Interchange in Liangjiang New District, Chongqing. The data were captured using a DJI Phantom 4 UAV, with a video resolution of 4096 × 2160 and a sampling rate of 30 fps. Vehicle trajectories were extracted from the video, as shown in Figure 2.

UAV videos are affected by wind disturbances, lighting variations, and fluctuations in flight attitude, which may introduce anomalies in the raw trajectory data such as sudden position jumps, vehicle ID switches, false detections, or missed detections. To ensure the reliability of subsequent interaction feature computation and risk assessment, systematic preprocessing of the trajectory data is required. The extracted data mainly include vehicle ID, spatial position, timestamp, frame number, and vehicle dimensions, comprising approximately twenty fields. The core information is derived from vehicle positions and time-series data. The meanings of the fields are presented in Table 1.

#### 2.1.2. Trajectory Data Processing

To ensure the physical and kinematic consistency of the trajectory data, systematic data quality control and trajectory reconstruction were applied to the raw trajectories, including removal of invalid trajectories, trajectory smoothing, coordinate transformation, and correction of kinematic anomalies.

- Invalid trajectory removal: Invalid trajectories were identified and removed based on predefined criteria related to spatial dispersion, motion characteristics, and observation duration. This step eliminates stationary objects, false detections, and incomplete trajectory segments to improve dataset reliability.
- Trajectory smoothing: Trajectory noise caused by detection errors and environmental disturbances was reduced using standard smoothing techniques. Abnormal stagnation segments were identified and corrected to maintain trajectory continuity.
- Coordinate transformation: Trajectory data were transformed from image coordinates into physical coordinates based on calibrated road geometry. Missing data points were interpolated to ensure temporal consistency across the trajectory sequence.
- Kinematic consistency correction: Velocity and acceleration values derived from the trajectory were screened for unrealistic fluctuations. Outliers exceeding reasonable physical limits were corrected using interpolation and smoothing to maintain physically plausible motion patterns.

### 2.2. Identification Framework

#### 2.2.1. Framework Overview

To ensure that risk identification results accurately reflect vehicle decision-making processes, this study develops a risk identification framework oriented toward decision-consistent evaluation. While maintaining model performance, the framework emphasizes the stability and generalizability of the identification results. It consists of three key stages:

Feature Construction: Only trajectory information observable prior to the decision moment is used, preventing leakage of future behavior information;

Risk Definition: Duration constraints are introduced to reduce the influence of transient fluctuations on risk determination;

Model Evaluation: A grouped data partitioning strategy is employed to assess model performance under different data conditions, enhancing the objectivity of generalization evaluation.

The core advantage of this framework lies in the phase separation mechanism between observation and conflict. By using kinematic game features from the ramp preparation stage to predict cpTTC risk levels in the actual merging stage, information leakage from labels to features is physically and logically eliminated, ensuring the deployability of the model in real-world autonomous driving warning systems.

#### 2.2.2. Interaction Feature Engineering

- Interaction Identification

Candidate Opponent Selection: Within the game time window $\left[t_{\mathrm{start}},\;t_{\mathrm{end}}\right]$, only mainline vehicles satisfying the following conditions are selected as candidate opponents: they are located within the target merging lane, their longitudinal distance to the agent is within a limited perception range, and their spatial position may lead to a potential insertion conflict. This step excludes distant vehicles or vehicles outside the target lane that are unrelated to the current merging decision, thereby narrowing the scope of the game participants.

Longitudinal Gap and Conflict Risk Characterization: To describe the relative relationship between the agent and candidate opponents, longitudinal gap and Time-to-Collision (TTC) are adopted as core indicators. The longitudinal gap is defined as:

(1) $$g_{\mathrm{long}}=x_{\mathrm{target}}-x_{\mathrm{agent}}$$

where $g_{\mathrm{long}}$ denotes the longitudinal gap between the target vehicle and the agent; $x_{\mathrm{target}}$ and $x_{\mathrm{agent}}$ denote the longitudinal positions of the target vehicle and the agent, respectively. Positive values indicate that the target vehicle is located ahead of the agent. Potential conflict risk is characterized by TTC extrapolated from short-term trajectories, reflecting the urgency of a possible collision if both vehicles maintain their current motion:

(2) $$p_{i}\left(t\right)=\;p_{i}+v_{i}e_{i}t$$

(3) $$TTC=min\{t>0:d(t)\leq d_{safe}\}$$

where $p_{i}(t)$ denotes the position vector of vehicle i at time t; $p_{i}$ denotes the initial position vector; $v_{i}$ denotes the vehicle speed; $e_{i}$ denotes the unit direction vector of motion; d(t) denotes the distance between two vehicles at time t; and $d_{safe}$ denotes the predefined safety distance threshold. If no potential collision exists, TTC is set to +∞.

Threat Scoring and Selection of Key Opponents: Among the candidate opponents, a comprehensive threat score is constructed to select the vehicle posing the greatest threat to the agent at each time step. The comprehensive threat score consists of distance-based threat, TTC-based threat, risk amplification factor, merging urgency weight, and the final score used for key opponent selection.

(4) $$S_{d}^{\left(j\right)}=\max\left(0,\;1-\frac{\left|g_{\mathrm{long}}^{\left(j\right)}\right|}{R_{\mathrm{perc}}}\right)$$

where $S_{d}^{\left(j\right)}$ denotes the distance-based threat score of candidate vehicle j; $g_{\mathrm{long}}^{\left(j\right)}$ denotes the longitudinal gap between the candidate vehicle and the agent; and $R_{\mathrm{perc}}$ denotes the perception range.

(5) $$S_{ttc}^{\left(j\right)}=\left\{\begin{array}{c} 0,\;\;{TTC}^{\left(j\right)}=+\infty \;\\ \max\left(0,\frac{T_{ttc,max}-{TTC}^{\left(j\right)}}{T_{ttc,max}}\right),\mathrm{otherwise} \end{array}\right.$$

where $S_{ttc}^{\left(j\right)}$ denotes the TTC-based threat score of candidate vehicle j; ${TTC}^{\left(j\right)}$ collision value; and $T_{ttc,max}$ denotes the maximum reference TTC.

(6) $$\gamma^{\left(j\right)}=\left\{\begin{array}{c} 1.5,\;{TTC}^{\left(j\right)}\leq \tau_{ttc}\;\\ 1.0,\;\mathrm{otherwise} \end{array}\right.$$

where $\gamma^{\left(j\right)}$ denotes the risk amplification factor; and $\tau_{ttc}$ denotes the TTC threshold.

(7) $$u=\exp\left(-\alpha \frac{d}{D_{\mathrm{ref}}}\right)$$

where u denotes the merging urgency weight; d denotes the distance from the agent to the merging point; $D_{\mathrm{ref}}$ denotes the reference distance; and α denotes the decay coefficient.


(8) $${\mathrm{Score}}^{\left(j\right)}=\left(\omega_{1}\;S_{d}^{\left(j\right)}+\omega_{2}\;\;S_{\mathrm{ttc}}^{\left(j\right)}\right)\cdot \gamma^{\left(j\right)}\cdot u$$

At each time step, the candidate vehicle with the highest comprehensive threat score is selected as the key interaction opponent:

(9) $$j^{*}=\arg\underset{j}{\max}\;{\mathrm{Score}}^{\left(j\right)}$$

where ${\mathrm{Score}}^{\left(j\right)}$ denotes the comprehensive threat score of candidate vehicle j; $\omega_{1}$ and $\omega_{2}$ denote the weighting coefficients satisfying $\omega_{1}$ + $\omega_{2}$ = 1. and $j^{*}$ denotes the selected key interaction opponent.

Key Interaction Opponent Determination. A selected opponent is considered to enter a substantive interaction with the agent when either the spatial distance or the collision risk reaches a critical level, defined as:

(10) $$\left|g_{\mathrm{long}}^{\left(j^{*}\right)}\right|\leq d_{\mathrm{key}}\mathrm{or}{\mathrm{TTC}}^{\left(j^{*}\right)}\leq \tau_{\mathrm{ttc}}$$

where $d_{\mathrm{key}}$ denotes the critical distance threshold. To validate the effectiveness of the interaction opponent identification method, a subset of typical merging events was manually checked using the original video, and the identified results were compared with the actual vehicle interactions. The verification results show that this method can reliably identify key interactive opponents in typical traffic scenarios, showing good rationality and stability.

- No-Leak Feature Engineering

In order to ensure the causal consistency in the process of identification and risk assessment of ramp merger strategies, this study applies no leakage constraints in the feature construction stage. The input characteristics of each training sample only come from the trajectory information that can be observed before the decision-making moment, and do not include any post-decision behavior or final result. Incorporating future frame information, such as deceleration, yielding behavior, or merge completion, may improve model performance during training; however, such information is not available in real-world deployment, which may introduce interpretation bias and reduce generalization capability. Therefore, each interaction event is represented by sequential observations within a pre-decision window, and dual-space interaction indicators are constructed in both Cartesian and Frenet coordinates to form event-level feature inputs.

The pre-decision observable window is defined as follows: for each ramp merging interaction event, the game start is $t_{0}$ with corresponding video frame $f_{0}$, and the sampling frequency is FPS=30 Hz. A fixed decision window ΔT=2.0 s is applied, with the decision time defined as:


(11) $$t_{d}=t_{0}+\Delta T$$

(12) $$f_{d}=f_{0}+\Delta T\cdot FPS$$

The set of observable times for feature construction is:

(13) $$W_{pre}=\{t|t_{0}\leq t\leq t_{d}\}$$

where $t_{d}$ denotes the decision time, and $W_{pre}$ denotes the observable pre-decision window. If an event ends prematurely, $t_{d}$ is adjusted to $min\left(t_{d},t_{end}\right)$ to avoid referencing non-existent future observations.

To further control information boundaries, only trajectory frames that actually exist are used within the window; short-term missing data are interpolated within the window, without extrapolation beyond the decision time. If a key main-lane opponent is absent or cannot be reliably matched in a frame, its associated variables are recorded as missing values without introducing virtual reference vehicles. The fixed 2 s decision window is chosen to cover the driver’s perception–evaluation–decision process during the initial merging phase while avoiding post-decision behavior that carries outcome information.

Interaction Feature Construction: Within the pre-decision window $W_{pre}$, a two-level interaction indicator system is established. The first level consists of frame-level interaction states, describing the vehicle interactions at each moment. The second level consists of event-level aggregated features, converting sequential information into fixed-dimension inputs.

Frame-level Interaction States: Let the ramp vehicle be A and the key main-lane opponent be B, with positions and speeds ($x_{A}$,$y_{A}$,$v_{A}$) and ($x_{B}$,$y_{B}$,$v_{B}$), respectively. At each observable time $t\in W_{pre}$, the interaction indicators are constructed as:

(14) $$dist\left(t\right)=\sqrt{{\left(x_{B}\left(t\right)-x_{A}\left(t\right)\right)}^{2}}+\sqrt{{\left(y_{B}\left(t\right)-y_{A}\left(t\right)\right)}^{2}}$$

(15) $$\Delta v_{long}\left(t\right)=v_{A}\left(t\right)-v_{B}\left(t\right)$$

(16) $$T_{catch}\left(t\right)=\left\{\begin{array}{c} \frac{{\;gap}_{long}\left(t\right)}{\Delta v_{long}\left(t\right)},{\;gap}_{long}\left(t\right)>0,\Delta v_{long}\left(t\right)>0\;\;\\ +\infty ,\;\;\;\;\mathrm{otherwise} \end{array}\right.$$

where $dist\left(t\right)$ denotes the Euclidean distance between the ramp vehicle and its interaction opponent at time t. Where $\Delta v_{long}\left(t\right)$ denotes the longitudinal relative speed; positive values indicate that the ramp vehicle is approaching the opponen. where $T_{catch}\left(t\right)$ denotes the potential time for the ramp vehicle to catch up with the opponent under the current relative motion condition. The relative displacement is projected along the ego vehicle’s travel direction to obtain the longitudinal ${gap}_{long}\left(t\right)$ and lateral gap ${gap}_{lat}\left(t\right)$, consistent with merging semantics.

To capture potential conflicts at the merge point, let the merge conflict point be m, and define the conflict-point time difference indicator cpTTC(t):

(17) $$cpTTC(t)=|T_{A\to m}(t)-T_{B\to m}(t)|$$

(18) $$T_{A\to m}(t)=\frac{{dist}_{A\to m}(t)}{v_{A}\left(t\right)}$$

(19) $$T_{B\to m}(t)=\frac{{dist}_{B\to m}(t)}{v_{B}\left(t\right)}$$

where cpTTC(t) denotes the absolute time diference for the two vehicles to reach the merge conflict point; smaller values indicate higher potential conflict risk. ${dist}_{A\to m}(t)$ and ${dist}_{B\to m}(t)$ denote the remaining distances from vehicles A and B to the merge conflict point, respectively.

In addition, to represent relative vehicle positions within the road structure, vehicle trajectories are projected into the road centerline coordinate system (Frenet coordinates), yielding longitudinal position s and lateral offset d. Further indicators are constructed as:

(20) $$\Delta s(t)=s_{B}(t)-s_{A}(t)$$

(21) $$\Delta v_{s}(t)=v_{s,\;A}(t)-v_{s,B}(t)$$

while retaining the ego vehicle’s lateral speed $v_{d,A}(t)$ to describe lateral motion intensity and potential merging intent.

Aggregated Event-level Features: To satisfy the fixed-dimension input requirement of machine learning models, sequential variables within the pre-decision window $W_{pre}$ are aggregated statistically. All features are computed within the 2.0 s pre-decision observable window. Key kinematic variables of the ego and interacting vehicles, such as longitudinal/lateral relative distance, relative speed, and acceleration, are summarized by mean, extrema, and standard deviation to represent the vehicles’ initial merging interaction state and intent. For trend-type variables, such as longitudinal gap and relative speed, both mean and standard deviation are retained to capture overall levels and fluctuations. For behavior-type variables, such as lateral speed, maxima or means are used to reflect whether significant merging maneuvers occur.

To capture short-term aggressive maneuvers, local-time-window statistics are introduced within the decision window. For example, the minimum acceleration is computed within a 1 s local window, and the relative minimum acceleration between the interacting vehicles is defined as:

(22) $$\Delta a_{min,local}=a_{min,local}^{\left(B\right)}-a_{min,local}^{\left(A\right)}$$

where $\Delta a_{min,local}$ denotes the difference in local minimum acceleration between the opponent and the ramp vehicle, which is used to characterize short-term defensive or forcing behavior during interaction.

#### 2.2.3. Risk Label Definition

- Risk Indicator and Threshold Setting

To enable proactive risk recognition, the evaluation interval for risk labels is strictly separated from the feature extraction interval $W_{pre}$. The actual interaction conflict period following the decision point $t_{d}$ is defined as the risk assessment window: $W_{post}=\{t|t_{d}<t\leq t_{end}\}$, where $t_{end}$ is the end time of the merging interaction event. The final event-level risk is determined solely based on the evolution of cpTTC within $W_{post}$, with the three-level risk classification as follows:

Safe: The time difference to the conflict point is sufficiently large, providing adequate reaction space, defined as cpTTC≥2.0.

General Risk: The time difference is moderate, indicating potential conflict requiring driver adjustment, defined as 1.0 s≤cpTTC<2.0 s.

High Risk: The time difference is small, with conflict potentially occurring imminently, defined as cpTTC<1.0 s.

- Duration Constraint for Risk State Determination

In actual trajectory data, measurement errors or short-term speed fluctuations may cause cpTTC to vary instantaneously between frames. Using a single-frame threshold alone may induce frequent state switches, reducing label stability and affecting model training. To address this, a duration constraint is applied: a risk state is assigned only if the threshold condition is met continuously for at least a predefined duration, set uniformly as: duration threshold=0.5.

This timescale effectively filters noise caused by transient fluctuations while preserving the genuine evolution of risk. To further reduce instability at boundaries, isolated risk segments shorter than the duration threshold are merged or ignored, maintaining temporal continuity of the final labels. Event-level risk labeling is thus defined as:

- cpTTC < 1.0 s and duration ≥ 0.5 s → High Risk.
- s ≤ cpTTC < 2.0 s and duration ≥ 0.5 s → General Risk.
- Otherwise → Safe.

Key parameters are set based on commonly reported ranges in traffic safety and driver behavior studies and are further aligned with the temporal characteristics of the dataset used in this research. The pre-decision observation window of 2 s corresponds to typical driver perception and reaction time (approximately 1.0–2.5 s) and is consistent with the observed duration of vehicle interaction processes in the collected trajectory data. The duration threshold of 0.5 s is introduced to suppress the influence of short-term fluctuations and to ensure that identified risk states reflect sustained interaction conditions rather than momentary disturbances. Critical time-gap thresholds of 1.0 s and 2.0 s are used to distinguish high-risk, general-risk, and safe states. These values are consistent with widely adopted temporal criteria in traffic conflict and surrogate safety analysis studies [30,31,32,33] and were observed to provide stable risk classification behavior under the traffic conditions represented in the dataset.

#### 2.2.4. Generalization-Oriented Model Evaluation

- Grouped Data Partition Strategy

In multi-vehicle ramp merging games, the kinematic features of the ego vehicle and interacting main-lane vehicles, such as relative distance and relative speed, are highly coupled and mirror each other. Random partitioning or splitting by individual agents can cause vehicles from the same game interaction to fall separately into training and test sets, resulting in severe event-level data leakage. To eliminate this risk, a strict “game pair”-based grouping strategy is adopted, binding the ego vehicle and its corresponding main-lane opponent as an inseparable pair and constructing a unique interaction group identifier:

(23) group_id=dataset ∥ interaction_id

where dataset denotes the data source or dataset version, and interaction_id denotes the unique identifier for a specific merging game pair.

In the process of GroupKFold cross-verification and data division, group_id is strictly used as a grouping constraint. This ensures that all samples from the same combined game event are regarded as an inseparable single unit and are completely and uniquely assigned to the training set or test set. This double-level isolation—including both the physical level and the event level—can effectively prevent the leakage of strongly coupled features between datasets, thus ensuring that the evaluation of the generalized performance of the model can be carried out accurately and objectively.

- Class Imbalance Handling

Risk identification datasets often show an unbalanced category distribution. Training directly based on the original sample may lead to the optimization process being dominated by the majority, thus reducing the ability to detect key risk states. To solve this problem, we adopt a loss function based on category weight:

(24) $$L_{weighted}=\sum_{i=1}^{N}\omega_{y_{i}}\cdot l\left(y_{i},{\hat{y}}_{i}\right)$$

where $\omega_{y_{i}}$ denotes the weight assigned to class $y_{i}$, and $l\left(\cdot \right)$ denotes the base loss function. Higher weight is assigned to the general-risk class to strengthen learning of the rare class, improving model stability under imbalanced conditions without altering the original data distribution.

- Evaluation Metrics

To comprehensively assess the performance of multi-class risk recognition models, a multi-dimensional metric system is established, covering both classification accuracy and probability quality.

Accuracy: Measures the overall proportion of correct predictions.


(25) $$Accuracy=\frac{TP+TN}{TP+TN+FP+FN}$$

F1 Score: Balances Precision and Recall.


(26) $$F1=\frac{2\cdot Precision\cdot Recall}{Precision+Recall}$$

Macro F1: Average F1 across all classes in multi-class tasks.


(27) $${F1}_{macro}=\frac{1}{K}\sum_{k=1}^{K}{F1}_{k}$$

Brier Score: Measures the error between predicted probabilities and true labels.

(28) $$Brier=\frac{1}{N}\sum_{i=1}^{N}\sum_{k=1}^{K}{{(p}_{ik}-y_{ik})}^{2}$$

where $p_{ik}$ denotes the predicted probability that sample i belongs to class k, and $y_{ik}$ is the one-hot encoding of the true label. The Brier score evaluates the reliability of model probability outputs and is commonly used in risk prediction and safety decision-making.

#### 2.2.5. Model Selection

In this study, three representative machine learning models were selected, each leveraging distinct learning principles and offering complementary strengths. Logistic Regression provides a simple and interpretable framework suitable for approximately linear relationships. Random Forest captures complex nonlinear interactions and is robust to noise, while XGBoost further enhances predictive accuracy through iterative residual fitting and regularization, making it particularly effective for challenging classification tasks such as traffic risk recognition, see Table 2.

Multinomial logistic regression models the probability that a sample belongs to class k given an input feature vector $x=(x_{1},x_{2},\ldots ,x_{d})$.

(29) $$P(y=k|x)=\frac{exp(w_{k}^{T}x+b_{k})}{\sum_{j=1}^{K}exp(w_{j}^{T}x+b_{j})}$$

where $w_{k}$ is the weight vector for class *k*, $b_{k}$ is the bias term, and K is the number of classes.

Random Forest aggregates predictions from *M* decision trees constructed on bootstrapped samples:

(30) $$\hat{y}=arg{max}_{k}\frac{1}{M}\sum_{m=1}^{M}I(T_{m}(x)=k)$$

where $T_{m}(x)$ is the prediction of the m-th decision tree, and I(⋅) is the indicator function. By aggregating multiple weak learners.

XGBoost minimizes a regularized objective by iteratively fitting residuals:

(31) $$L=\sum_{i=1}^{N}l\left(y_{i},{\hat{y}}_{i}\right)+\sum_{k=1}^{K}\Omega \left(f_{k}\right)$$

where l(⋅) is the loss function, $f_{k}$ denotes the k-th tree model, and $\Omega \left(f_{k}\right)$ is a regularization term controlling model complexity.

## 3. Results

### 3.1. Performance Comparison of Multiple Models for Risk Identification

To evaluate the suitability of different models in risk identification, Logistic Regression, Random Forest, and XGBoost were compared. Considering the extreme sparsity of the “Class 1—General Risk” samples, class weight strategies were applied during training, and Macro-average and Balanced Accuracy were used as primary evaluation metrics. Table 3 presents the evaluation results based on the event-level grouped test set.

The results indicate that XGBoost achieves the best overall performance, with the highest Macro-average F1 (0.5830) and Macro-average AUC (0.8898), as well as the lowest Brier score (0.1964), demonstrating the most reliable probability calibration. Logistic Regression performs the weakest and struggles to capture complex nonlinear interaction features. Notably, model performance exhibits significant imbalance. Although Random Forest achieves the highest overall Accuracy (0.8232), its Balanced Accuracy drops to 0.5143, substantially lower than XGBoost (0.6516), indicating that its high accuracy relies mainly on majority-class fitting and provides almost no recall for the extremely sparse and boundary-ambiguous “Class 1” samples. It should also be noted that the absolute value of the Macro-average F1 remains at a moderate level, reflecting the inherent difficulty of multiclass risk identification under highly imbalanced natural driving data conditions. Overall, XGBoost demonstrates the strongest comprehensive and balanced discrimination capability under extreme class imbalance and is therefore selected as the core reference model for subsequent analyses.

### 3.2. Model Stability Analysis

To evaluate the generalization capability of models in entirely new traffic interaction scenarios and to completely prevent event-level data leakage between game vehicles, a GroupKFold cross-validation strategy constrained by “Game Pair” was employed for stability analysis. Table 4 presents the performance of each model under cross-validation.

Overall, XGBoost achieves the same Accuracy (0.8582) as Random Forest but with a smaller standard deviation (±0.0267), and attains the highest mean values in Balanced Accuracy (0.5746), F1-macro (0.5796), and AUC (0.8701), demonstrating the strongest robustness on unseen data partitions.

It is worth noting that tree-based models (Random Forest and XGBoost) exhibited relatively high standard deviations (about ±0.11) in equilibrium accuracy and F1-macro metrics. This variability is not caused by algorithmic instability, but rather by the inherent challenges of the dataset, particularly the limited representation of the General Risk category. In cross-validation, the scarcity of General Risk events in each subset can lead to fluctuations in recall and F1 metrics for this class, amplifying the variance of macro-average indicators.

In short, after taking into account the statistical uncertainty caused by sparse categories, the model maintains a high degree of stability in the core task. The game-based cluster cross-verification of groups can effectively prevent the phenomenon of human exaggeration of performance due to feature coupling, thus providing an objective and reliable benchmark for evaluating the generalization ability of the model in actual deployment.

### 3.3. Evaluation of Predictive Probability Reliability

In the traffic risk early warning system, the reliability of the model prediction probability is directly related to the effectiveness of decision-making support. Excessive confidence or lack of confidence in a specific risk state may lead to serious false alarms or missed tests. Therefore, this study uses multiple types of Brier scores to quantify the quality of probability calibration. The lower the Brier score, the closer the probability distribution of the prediction is to the observed risk occurrence, reflecting the higher reliability of the probability prediction, see Table 5.

As shown in Table 4, the models exhibit differences in probability calibration. XGBoost achieves the lowest Brier Score on the test set (0.1964), indicating the predicted probability distribution aligns most closely with the observed risk states and can provide an objective basis for decision adjustments based on probability thresholds. Random Forest performs moderately (0.2194). In contrast, Logistic Regression exhibits a higher Brier Score (0.3466), suggesting that the linear model produces less reliable confidence estimates when representing complex traffic interaction features. Overall, the calibration results further confirm that XGBoost not only excels in classification performance but also provides risk probability outputs with higher quantitative reliability.

### 3.4. Investigation of Key Risk Factors

To examine the identification bottleneck of Class 1—General Risk in multi-class tasks—permutation feature importance was employed to assess the contribution of features in distinguishing Class 1 from Class 0—Safe and Class 2—High Risk. This method quantifies the actual impact of each feature by randomly shuffling its values and observing performance degradation, as shown in Table 6.

The distinguishability between Class 1 and Class 2 (AUC = 0.7741) is higher than the distinguishability between Class 1 and Class 0 (AUC = 0.6531), which indicates that the General Risk and security state overlap in the characteristic space is high, which blurs Decision-making boundaries. In the replacement analysis of Class 1 vs. Class 0, the importance of most features is close to zero, and only ${gap}_{lat}^{std}$ shows a weak response (about −0.02 ± 0.06). This confirms that a single characteristic disturbance alone is not enough to change the model decision-making from a “Safe” state to a “General Risk” state, reflecting the strong homogeneity in kinematic characterization. In contrast, multiple characteristics have shown a significant distinguishing effect in distinguishing between Class 1 and Class 2. The first ten characteristics have been listed in Table 7.

In general, the analysis of arrangement importance reveals the inherent law of risk distribution in physical space: Class 1—General risk represents a transitional state that tends to be “safe”, and its high difficulty in identification is mainly due to the overlap of inherent characteristics rather than the lack of model fitting ability. This insight provides data support for the summary of risk levels and the setting of early warning thresholds in the autonomous driving system.

## 4. Discussion

### 4.1. Physical Scene Constraints and Temporal Generalization Task Reconstruction

The dataset in this study was collected from typical ramp merging scenarios in mountainous cities, characterized by short merge zones, uneven lane numbers, and large speed differences. The data are divided into different time slices—masked_1, masked_2, and masked_3—corresponding to distinct peak traffic periods.

Under these extreme spatiotemporal constraints, high-frequency game interactions and conflicts (Risk states) are the macro-norm, while micro-level General Risk (Class 1) features are prone to fragmentation across different temporal subdomains. In cross-domain evaluation, small sample sizes can cause dramatic fluctuations in metrics. It should be clarified that the two-class formulation introduced in this section is not intended to redefine the original risk taxonomy, but rather to serve as a diagnostic task to evaluate model stability under extreme temporal imbalance conditions. Table 8 compares the stability of metrics under different task dimensions.

As shown, reducing the task to a two-class problem (Safe vs. Risk) significantly improves cross-period evaluation stability (standard deviation converging to ±0.0215).

### 4.2. Class-Wise Analysis in Extreme Temporal Imbalance

During peak periods with extreme class imbalance, models are prone to the “majority class trap (Accuracy Paradox),” predicting the majority class excessively and inflating overall accuracy. Table 9 shows the best and worst temporal transfer scenarios, with class-wise recall validating model decision logic.

Table 8 illustrates underlying temporal representation differences. In the worst-case scenario, Logistic Regression fails to handle nonlinear shifts caused by traffic flow changes, degenerating into a “full risk predictor”, with F1-macro falling to 0.4936. In contrast, XGBoost maintains Risk Recall (0.9978) while correctly identifying approximately 70% of minority Safe samples (Safe Recall = 0.6923), demonstrating consistent decision behavior across temporal domains with different traffic density levels, rather than relying on a specific distribution of traffic states.

### 4.3. Ablation Analysis of Class Weights

To address structural class imbalance across temporal evolution, cross-domain ablation experiments on Class Weight were conducted to evaluate the necessity of explicit weighting. Table 10 presents the results.

The results show that explicit class weighting has minimal effect on XGBoost (−0.0020) but substantially harms Logistic Regression (−0.0870). This indicates that XGBoost’s tree-splitting gains and gradient optimization inherently accommodate minority class boundaries across peak periods, reducing the dependence on explicit external weighting while addressing long-tail distributions, enhancing practical deployment feasibility.

### 4.4. Limitations and Future Work

Feature extraction depends on predetermined observation data, which needs to be consistent with the actual system input constraints and provide a quantitative basis for risk classification. However, there are still some limitations: the data mainly comes from a single ramp merger scenario and does not cover a more complex road network structure; risk tags are set based on thresholds and cannot capture the continuity of risk evolution. In addition, the boundary between General Risk and adjacent risk states may become unstable under extreme temporal imbalance conditions, which partially explains the observed variability in three-class performance.

Future work will expand data sources and explore the use of time series characteristics for dynamic risk modeling to enhance its applicability in complex traffic environments. In addition, future studies will incorporate multi-condition datasets, including different weather scenarios and day–night traffic operations, to further examine their influence on driver behavior and risk formation. Future research will also consider driver heterogeneity factors, such as age-related differences in reaction time and decision-making characteristics, to better understand their potential impact on interaction dynamics and risk recognition performance.

## 5. Conclusions

This study develops a decision-consistent risk recognition framework for vehicle interactions in short weaving sections of mountainous city expressway. The main findings can be summarized as follows.

First, a decision-consistent interaction feature system is established by restricting model inputs to pre-decision trajectory information within a defined observation window. This design reduces the risk of information leakage and ensures that the feature construction process is aligned with realistic decision conditions. The experimental results demonstrate that the proposed feature system supports stable multi-class risk recognition under real traffic interaction scenarios.

Second, the comparative analysis of Logistic Regression, Random Forest, and XGBoost models indicates that traffic risk formation exhibits nonlinear characteristics. Among the evaluated models, XGBoost shows relatively better overall classification performance and probability calibration stability, as reflected by its lower Brier score and consistent performance under imbalanced data conditions. These results suggest that ensemble learning methods are suitable for capturing complex interaction patterns in weaving traffic environments.

Third, temporal cross-domain evaluation reveals that the proposed framework maintains consistent recognition behavior under varying traffic demand conditions. The observed performance stability suggests the existence of relatively invariant micro-level interaction patterns in vehicle behavior, which supports the general applicability of the decision-consistent modeling strategy across different traffic states.

## Figures and Tables

**Table 1:** Key Fields and Their Descriptions in the Raw Dataset.

Field	Description
frameNUM	Frame number, recorded at a frame rate of 30 frames per second
carID	Vehicle ID, uniquely and consistently assigned to each vehicle throughout the dataset
carCenterX	Lateral coordinate of the vehicle center in the local coordinate system, referenced from the bottom of the frame
carCenterY	Longitudinal coordinate of the vehicle center in the local coordinate system, referenced from the start of the left section of the road
pix	i = 1, 2, 3, 4, x-coordinates of the vertices of the vehicle detection box

**Table 2:** Comparison of the Three Models.

Model	Core Principle	Strengths	Limitations
Logistic Regression	Probabilistic discriminative model	Simple, interpretable, fast training, works well for linearly separable data	Limited capacity for nonlinear relationships
Random Forest	Ensemble of decision trees (Bagging)	Captures nonlinear patterns, robust to noise and overfitting, stable predictions	Less interpretable, can be computationally heavier
XGBoost	Gradient Boosting Trees with regularization	High predictive accuracy, handles complex nonlinearities, mitigates overfitting	Requires careful parameter tuning, more computationally intensive

**Table 3:** Event-level test-set performance of risk identification models.

Model	Accuracy	Balanced Accuracy	F1-Macro	AUC-OvR-Macro
XGBoost	0.8182	0.6516	0.5830	0.8898
RandomForest	0.8232	0.5143	0.5219	0.8763
LogisticRegression	0.7323	0.4189	0.4443	0.7749

**Table 4:** Stability Results of GroupKFold Cross-Validation.

Model	Accuracy	Balanced Accuracy	F1-Macro	AUC-OvR-Macro	Brier
LogisticRegression	0.7772 ± 0.0308	0.4801 ± 0.0837	0.4577 ± 0.0334	0.7492 ± 0.0893	0.3208 ± 0.0402
RandomForest	0.8582 ± 0.0306	0.5646 ± 0.1132	0.5765 ± 0.1183	0.8544 ± 0.0572	0.2186 ± 0.0338
XGBoost	0.8582 ± 0.0267	0.5746 ± 0.1104	0.5796 ± 0.1088	0.8701 ± 0.0476	0.2220 ± 0.0495

**Table 5:** Comparison of Brier Scores for Predicted Probabilities Across Models.

Model	Brier Score (Test Set)
XGBoost	0.1964
RandomForest	0.2194
LogisticRegression	0.3466

**Table 6:** Pairwise Identification Performance of Class 1 with Safe and High Risk.

Pairwise Task	F1	AUC
Class 1 vs. Class 0	0.2500	0.653061
Class 1 vs. Class 2	0.1818	0.774138

**Table 7:** Top Features for Distinguishing General Risk from High Risk.

Feature	$v_{agent}^{max}$	$a_{long}^{max}$	${\bar{a}}_{long}$	${dist}_{A\to m}^{std}$	$v_{agent}^{std}$	${gap}_{lat}^{std}$	${∆v}_{long}^{std}$	$v_{agent}^{min}$	$v_{d,agent}^{max}$	${gap}_{lat}^{max}$
importance	0.1170	0.0956	0.0919	0.0281	0.0186	0.0170	0.0135	0.0104	0.009	0.008

**Table 8:** Impact of Task Dimensions on Metric Stability.

Task Architecture	Cross-Domain F1-Macro Range	Metric Std
Original Three-Class	0.5426~0.8548	±0.1432
Reconstructed Two-Class	0.8390~0.8886	±0.0215

**Table 9:** Class-wise Recall in Typical Temporal Cross-Domain Transfer Scenarios (Two-Class).

Performance	Model (Temporal Transfer)	Safe Recall	Risk Recall	F1-Macro
Best Generalization	XGBoost (masked_2 → masked_3)	0.6923	0.9978	0.8886
Worst Generalization	LogisticRegression (masked_2 → masked_1)	0.0000	1.0000	0.4936

**Table 10:** Ablation Results of Class Weights.

Model	No Weight F1-Macro	with Weight F1-Macro	ΔF1
LogisticRegression	0.7161	0.6291	−0.0870
Random Forest	0.8366	0.8393	+0.0027
XGBoost	0.8595	0.8575	−0.0020

## Data Availability

The raw data supporting the conclusions of this article will be made available by the authors on request.
